# Digital Divide: Contrasting Provider and User Insights on Healthcare Services During the COVID-19 Pandemic

**DOI:** 10.3390/healthcare13151803

**Published:** 2025-07-25

**Authors:** Olympia Anastasiadou, Panagiotis Mpogiatzidis, Katerina D. Tzimourta, Pantelis Angelidis

**Affiliations:** 1 Nursing Department, General Hospital of G. Gennimatas, 54635 Thessaloniki, Greece; 2 Department of Electrical and Computer Engineering, University of Western Macedonia, 50100 Kozani, Greece; 3 4th Healthcare Authority of Greece, 54695 Thessaloniki, Greece; bogiatzidis@yahoo.com; 4 Department of Midwifery, School of Health Sciences, University of Western Macedonia, 50200 Ptolemaida, Greece; 5 Laboratory of Biomedical Technology and Digital Health, Department of Electrical and Computer Engineering, University of Western Macedonia, 50100 Kozani, Greece; ktzimourta@uowm.gr (K.D.T.); paggelidis@uowm.gr (P.A.)

**Keywords:** digital divide, crisis, COVID-19, healthcare services

## Abstract

Introduction: This prospective descriptive study explored the disparities in perceptions and experiences regarding healthcare services between providers and users during the COVID-19 pandemic, with a specific focus on the impact of the digital divide on access to and quality of care. The study revealed significant inconsistencies in the experiences of healthcare providers and patients, particularly regarding the effectiveness of digital health interventions. Methods: This study was a prospective descriptive analysis conducted to evaluate and compare the use of electronic healthcare services between healthcare employees (HΕs) (N = 290) and consumers (Cs) (N = 263) from December 2024 to May 2025, utilizing an electronic survey after the COVID-19 pandemic. To ensure the statistical validity of the sample size, a power analysis was performed using G*Power 3.1.9.2 software. A questionnaire was developed to evaluate the readiness of healthcare employees and consumers for electronic healthcare services. It was validated to ensure reliability within this population and comprised 49 questions. Results: The response rate of the participants was 89.19%, and the Cronbach’s alpha for the questionnaire was 0.738. The study revealed notable differences in perceptions regarding health-related information and digital health technologies across genders and age groups. Specifically, 28.8% of females and 27.3% of males considered it important to be well-informed about health issues (χ^2^ = 8.83, df = 3, *p* = 0.032). Conclusions: This research contributes to filling a gap in comparative analyses of provider and user perspectives, offering a comprehensive view of how digital health was adopted and experienced during a global crisis. Practically, it provides an evidence base to guide future interventions aimed at fostering more equitable, resilient, and user-friendly digital healthcare systems.

## 1. Introduction

In an increasingly digital world, the intersection of technology and healthcare has garnered significant attention, particularly in light of unprecedented challenges faced during the COVID-19 pandemic. The pandemic has acted as a catalyst for healthcare providers and consumers alike to re-evaluate their approaches toward digital healthcare services, emphasizing the critical role of technology in ensuring access to necessary medical care. This transformation in healthcare delivery highlights the disparities known as the digital divide, a term that encapsulates the gap between those who have easy access to digital technology and those who do not. Researchers have thus begun to explore the multifaceted dimensions of this divide, especially in relation to how it affected patient–provider interactions during this health crisis [1]. The significance of understanding these dynamics lies not only in addressing immediate healthcare needs but also in improving long-term healthcare policies and practices in a post-pandemic world.

The existing literature reveals a myriad of themes surrounding the digital divide within healthcare contexts, from disparities in technology access to varying perceptions of telehealth effectiveness. For instance, one study found that patients in lower socioeconomic brackets experienced significant challenges in utilizing telehealth services, which were otherwise deemed effective by their more affluent counterparts [2]. This theme of socioeconomic disparity, intertwined with issues of technology literacy, emerged repeatedly in studies, indicating that while certain groups adapted seamlessly to online healthcare services, others struggled, often due to an inadequate digital infrastructure or a lack of digital skills [3,4]. Furthermore, healthcare providers have reported their perspectives on the urgency for technological integrations that could potentially bridge these gaps, though they often lack comprehensive training on how to effectively engage marginalized populations through digital platforms [5,6].

Insights from providers during this period often reflected a focus on technological implementation rather than addressing user-specific barriers, suggesting a disconnect between supply and demand in digital health services [7]. Research indicated that many healthcare providers began to recognize the necessity of integrating user feedback into their digital service offerings. This was corroborated by findings that illustrated a growing awareness among practitioners of the unique challenges faced by patients, such as a lack of internet access and digital literacy, which exacerbated the divide [8,9]. Qualitative studies serve as a critical lens, illuminating the nuanced experiences of healthcare providers and patients alike. For instance, interviews conducted with healthcare workers illuminated the barriers they faced in delivering telehealth services, emphasizing a technological and infrastructural gap that disproportionately affected low-income populations [10]. Conversely, user testimonies highlight the differing levels of digital literacy, suggesting that the pandemic exacerbated existing disparities in access to healthcare resources [8].

In reality, users encounter considerable obstacles, such as limited access to devices, unreliable internet connectivity, and gaps in digital literacy. Research conducted during the pandemic revealed that only 60% of patients had access to a smartphone, with an even smaller percentage possessing a computer or tablet. While providers commonly focus on the technical advancement of digital healthcare solutions—such as creating telemedicine platforms or mobile health apps—users tend to prioritize ease of use and accessibility. The usability challenges for users can include difficulty navigating systems, unclear instructions, and a lack of adequate support. Studies indicate that patients struggling with technical issues or poor platform design are less inclined to use these services again in the future [11].

Another discrepancy lies in the perceived advantages of digital healthcare. Healthcare providers frequently highlight convenience, efficiency, and cost savings, whereas users are more attuned to the quality of care, level of personalization, and human interaction these services provide. Surveys of telemedicine users during the pandemic showed an appreciation for convenience but also revealed dissatisfaction with the diminished personal connection compared to traditional in-person care. Socioeconomic and demographic factors further exacerbate this digital divide. Populations such as low-income groups, minorities, and residents in rural areas encounter significant barriers, contributing to existing health inequities [12].

Research during the pandemic underscored how unequal access to digital health perpetuates disparities in healthcare outcomes among these vulnerable populations. To bridge this divide, studies advocate for healthcare providers to embrace user-centered design principles. Digital services should be tailored to ensure they are accessible, intuitive, and responsive to all users’ needs [11,12,13].

However, while the literature elucidates these distinct user and provider insights, there remains a notable gap in comparative research that holistically examines both perspectives simultaneously. Most available studies tend to put greater emphasis on either the user experience or the providers’ views, which limits the understanding of the healthcare ecosystem in its entirety [14]. This gap indicates an urgent need for further investigations that juxtapose these contrasting insights to better inform healthcare policies, digital literacy programs, and resource allocations. Moreover, studies have often focused on short-term access issues rather than delving into long-term implications for patient care and health outcomes [15,16].

The aim of this study is to explore the varying perspectives of healthcare providers and users concerning the adoption and efficacy of digital health services during the COVID-19 pandemic, with specific emphasis on issues of accessibility and usability. To address these challenges, the research employs quantitative methodologies, including surveys and interviews with both providers and users, to capture their experiences and assess their levels of satisfaction with digital health solutions.

The main objectives of this investigation include analyzing user experiences with digital healthcare services alongside evaluating healthcare providers’ perceptions regarding the implementation of telehealth platforms. By systematically collecting and examining these experiences, the study aims to identify the underlying motivations, obstacles, and enabling factors encountered by both groups when utilizing healthcare services in the context of the COVID-19 pandemic.

## 2. Material and Methods

This study employed a prospective descriptive comparative analysis to assess and contrast the utilization of electronic healthcare services by healthcare employees (N = 290) and consumers (N = 263) over the period from December 2024 to May 2025, using data collected through an electronic survey. To ensure the robustness of the sample size, a power analysis was conducted with the G*Power 3.1.9.2 software. The analysis incorporated parameters such as the effect size (d = 0.2), significance level (α = 0.05), and statistical power (0.8), indicating that a total sample size of N = 620 was necessary to obtain statistically significant outcomes. This sample size was evenly distributed into two groups: healthcare employees (HEs, N = 310) and consumers (Cs, N = 310).

Participants were selected through purposive sampling to guarantee diversity in demographic backgrounds and varying degrees of experience with digital health in both groups. The target sample consisted of 310 healthcare employees and 310 consumers to enable statistically meaningful comparisons. Ultimately, valid responses were obtained from 290 HEs and 263 Cs.

The recruitment process was conducted electronically, utilizing emails and online health community networks as the primary channels. Healthcare professionals were engaged through targeted communication strategies that included internal hospital notifications and outreach via professional associations. Conversely, consumers were approached through collaborations with patient advocacy organizations, community health centers, and social media campaigns to ensure representation across diverse demographic groups. All prospective participants received detailed information regarding the objectives of the study and were assured of confidentiality protocols. Informed consent was obtained digitally prior to initiating the questionnaire. The survey instrument, comprising 49 validated items, was made available online and structured to be completed within approximately 20 min to optimize participants’ convenience and accessibility for individuals across a range of digital skill levels.

A questionnaire was developed to evaluate the readiness of healthcare employees and consumers for electronic healthcare services. It was validated to ensure reliability within this population and comprised 49 questions. Factor analysis did not reveal the presence of any subscales. The reliability of the questionnaire was measured, and a Cronbach’s alpha value was determined.

The validation of our new questionnaire was a thorough and structured process aimed at ensuring its reliability, accuracy, and effectiveness in measuring the intended concept. This multifaceted approach comprised several key phases, each designed to rigorously evaluate the instrument’s psychometric properties. The initial step focused on assessing face validity by reviewing whether the questionnaire items appeared relevant to the concept being measured. A panel of field experts provided valuable feedback, which guided refinements to ensure clarity, precision, and relevance in the items. This collaborative review laid the foundation for developing a robust instrument. Subsequently, a content validity study was conducted to confirm that all pertinent dimensions of the concept were adequately represented. A systematic framework was used to identify relevant domains and sub-domains, followed by the development of items targeting each aspect. A detailed literature review informed this process by incorporating insights from existing measures of the concept. Expert evaluations further ensured that each item was clear and accurately aligned with its intended dimension, solidifying the questionnaire’s comprehensiveness. The construct validity assessment involved administering the questionnaire to a participant sample and analyzing the relationships between its items and other relevant measures of the concept. These findings confirmed that the items effectively captured the essence of the concept and were theoretically consistent.

The study protocol received approval from the Scientific Committee of the University of Western Macedonia in Greece (ID: 586/16-12-2022). All participants provided informed consent at the beginning of the questionnaire, and their confidentiality was maintained throughout data collection and processing. The demographic data collected included gender, age, marital status, education level, knowledge of foreign languages, and years of work experience.

The statistical analyses were performed using SPSS 22 (IBM SPSS Software, Chicago, IL, USA). The Kolmogorov–Smirnov test was employed to assess normality. The comparisons were carried out using the chi-square test and Spearman’s rho, with the data expressed as the mean ± standard deviation and a significance level of 0.05.

## 3. Results

The response rate of the participants was 89.19%, and the Cronbach’s alpha for the questionnaire was 0.738. The demographic data of the HEs and Cs are shown in Table 1. In Table 2, we present the employment relationships of the HEs.

### Correlations Between the Questionnaire and the Demographic Data

There was no significant correlation between the HEs and Cs for the question, “How familiar are you with using a computer?” (χ^2^ = 7.42, *p* = 0.115). However, for the question, “How well do you know the following concepts such as username, password, settings, information system?” a significant difference was observed (χ^2^ = 10.58, *p* = 0.032). Specifically, 45.17% of HEs and 32.31% of Cs reported excellent familiarity with these terms.

Additionally, in response to the question, “I know how to stay informed about health issues,” 35.17% of HEs and 15.2% of Cs indicated to a very great degree that they knew how to stay informed (χ^2^ = 37.03, *p* < 0.001). For the question, “Have you used digital health applications during a period of crisis (pandemic, natural disasters, epidemics)?” 86.55% of HEs and 80.22% of Cs reported using healthcare applications during the COVID-19 pandemic (χ^2^ = 4.01, *p* = 0.05).

For the question, “Have you received training on the use of digital health tools?” 75.51% of HEs and 87.07% of Cs stated they had not received any training in using digital health tools. On the other hand, for the question, “How important do you consider the use of digital health services in times of crisis?” 59.65% of HEs and 47.14% of Cs regarded the use of digital health services during the COVID-19 pandemic as important (χ^2^ = 10.27, *p* = 0.036).

Finally, when asked about the financial impact through the question, “My treatment costs during the health crisis were zero using digital health applications,” 18.62% of HEs and 26.23% of Cs considered the lack of cost associated with digital health services during the COVID-19 pandemic as significant (χ^2^ = 49.18, *p* < 0.001).

The study revealed notable differences in perceptions regarding health-related information and digital health technologies across genders and age groups. Specifically, 28.8% of females and 27.3% of males considered it important to be well-informed about health issues (χ^2^ = 8.83, df = 3, *p* = 0.032). Among the females, 47% reported obtaining health information from various sources (χ^2^ = 8.73, df = 1, *p* = 0.004), while 17% believed they could safeguard their health through online resources (χ^2^ = 17.88, df = 4, *p* = 0.001). Additionally, 6.5% of males and 2.9% of females deemed it highly significant that digital health technologies fostered greater participation and empowerment in managing their health during the crisis (χ^2^ = 14.26, df = 4, *p* = 0.007). Furthermore, 12.25% of males and 9.8% of females expressed the view that their treatment costs during the health crisis were effectively reduced to zero through the use of digital health applications (χ^2^ = 45.59, df = 4, *p* < 0.001).

Age also played a role in the attitudes toward digital health technologies. Within the age group of 20–30 years, 3.1% expressed confidence in their ability to appropriately utilize digital health tools, whereas this sentiment was shared by 11.4% of individuals aged 41–50 years (χ^2^ = 27.1, df = 16, *p* = 0.04). Similarly, 3.1% of respondents aged 20–30 years and 11.4% of those aged 41–50 years emphasized the importance of healthcare facility staff accepting the integration of digital tools into practice (χ^2^ = 28.06, df = 16, *p* = 0.031).

## 4. Discussion

Contrasting providers’ and users’ insights on healthcare services during the COVID-19 pandemic, this study aimed to explore the divergence in experiences and perceptions of healthcare providers and users regarding digital health access during a period of rapid transformation. The paper’s core contribution lies in its novel juxtaposition of these two crucial perspectives, filling a gap in the literature and providing a more nuanced understanding of the digital divide beyond mere access statistics.

The transition to telehealth during the COVID-19 pandemic marked a significant shift in healthcare delivery, which is particularly illuminated by the insights gathered from both healthcare providers and users. The analysis revealed that while there was an overall increase in the adoption of digital platforms among healthcare providers, a notable digital divide persisted, greatly affecting access to services for various demographic groups [1]. The key findings indicated that users reported a significant improvement in communication and engagement with their healthcare providers through telehealth, yet they simultaneously experienced discomfort with technology and a lack of digital literacy, which hindered their overall experience [2]. Contrastingly, healthcare providers expressed optimism about the potential of telehealth, citing its efficiency in reaching patients and providing care while acknowledging concerns over digital inequities that have limited its effectiveness among underserved populations [17]. This divergence in experiences aligns with previous studies suggesting that socioeconomic factors can significantly influence digital health engagement and access [16].

From the healthcare providers’ perspective, digital health tools were largely perceived as efficient, cost-effective, and instrumental in sustaining patient care continuity amidst unprecedented disruptions. Many providers acknowledged telehealth’s capability to extend their reach while reducing physical contact and supporting operational resilience during lockdowns. However, their optimism was tempered by an acute awareness of the barriers faced by patients, especially those from vulnerable or underserved populations. Providers identified persistent hurdles, including digital literacy gaps, unreliable internet access, and insufficient user training, as significant factors limiting the efficacy and equity of telehealth services [18].

On the other hand, patients highlighted a distinct set of challenges associated with using digital health tools. While many appreciated the convenience and safety afforded by virtual consultations, technical challenges—such as poor usability, software glitches, and reduced interpersonal connection with healthcare professionals—were noted as sources of frustration. For certain demographic groups, particularly older adults, economically disadvantaged households, and rural residents, limited technological proficiency and inconsistent internet connectivity represented major obstacles to effective engagement with digital health services. These findings are congruent with broader research that underscores the influence of socioeconomic determinants on access to and satisfaction with digital health platforms [19].

The juxtaposition of these perspectives underscores a central principle: addressing the digital divide necessitates more than mere technological deployment. Achieving equitable digital health requires a multidimensional approach that involves tailoring solutions to diverse user needs, accommodating varying degrees of technological familiarity, and closing the infrastructural gaps that are prevalent in many communities. Effective policies and strategies should prioritize investments in user-centered design principles, accessible platform interfaces, comprehensive digital literacy programs for both providers and patients, and efforts to ensure universal access to reliable internet services [18,20].

In addition to offering practical insights, this study yields significant policy recommendations. To optimize the advantages of digital healthcare systems, future strategies must emphasize enhancing digital literacy, ensuring affordable access to devices and connectivity, and targeting interventions toward populations most susceptible to exclusion from the digital healthcare transition. Importantly, care must be taken to ensure that digital solutions complement rather than entirely replace traditional forms of healthcare delivery, thereby preserving the human connections that are vital to effective patient–provider interactions [21].

Academically, this research contributes substantively to bridging a key gap in comparative analyses of provider and user experiences with digital health technologies during a global crisis. It offers a nuanced understanding of how these tools were operationalized and perceived under challenging circumstances. On a practical level, it provides an empirical foundation upon which future interventions can be designed to foster equitable, resilient, and user-centric digital healthcare systems [13].

A key theme emerging from the results is the clear divergence in how providers and users experience digital healthcare. Healthcare providers largely viewed digital health solutions as necessary and efficient tools for maintaining patient engagement and continuity of care when in-person interactions were restricted. Many reported positive experiences with telehealth, citing its potential to reduce costs, improve scheduling flexibility, and expand services to remote areas. However, providers were also acutely aware of the systemic barriers that limit equitable access, particularly among socioeconomically disadvantaged groups [22].

For users, the experience was more nuanced. Many participants appreciated the convenience of remote consultations, especially during a time when physical distancing was essential. However, significant obstacles diminished these perceived benefits. Users reported difficulties with technology, including unfamiliar interfaces, complex login procedures, and unreliable internet connectivity. These challenges are consistent with previous research indicating that the usability of digital platforms strongly influences whether patients will adopt and continue using them. Moreover, the lack of personal interaction and the perceived impersonality of telehealth left some users dissatisfied, highlighting the enduring importance of the human connection in healthcare [20].

This study’s comparative approach addresses a gap in the literature, which has often focused on either provider readiness or user adoption in isolation. By examining both perspectives simultaneously, the research demonstrates that digital transformation in healthcare cannot succeed if it overlooks the complex realities of end-users. This calls for a shift from a purely technology-driven implementation to a more user-centered design and support model. Training programs tailored to varying literacy levels, clearer communication about how to use digital tools, and proactive outreach to underserved communities are essential steps toward bridging the digital divide [16].

Notably, the results aligned with existing literature that underscores the emergence of the digital divide during the pandemic, where high-income populations benefited considerably more from telehealth services than low-income individuals [5]. Furthermore, the findings revealed that barriers such as a lack of internet connectivity and inadequate training were common challenges faced by users, mirroring issues documented in prior research [6]. While providers highlighted adaptations in their practices, they also recognized the shortcomings in addressing the systemic barriers faced by users, which extends the findings from existing studies highlighting the necessity for comprehensive digital health policies that bridge these gaps [14].

This contrast between provider optimism and user challenges underscores the critical need for addressing the digital divide in healthcare, with implications for policy and practice. Addressing these disparities is imperative not only for improving health outcomes but also for fostering equitable access to care in future health crises [15]. The significance of this research extends to both academia and practical applications, emphasizing the importance of integrating user-centered approaches in telehealth strategies to support vulnerable populations. Thus, the findings provide a foundation for future research that can inform interventions aimed at enhancing digital literacy and access, thereby promoting a more equitable digital health landscape [23].

Overall, the findings contribute to a deeper understanding of how digital health can support resilient healthcare systems during crises while underscoring the urgent need to tackle structural inequities. Future studies should build on this work by exploring long-term outcomes of digital health adoption and by developing targeted strategies to promote inclusive and accessible care for all populations.

Although this research was carefully prepared, we are still aware of its limitations and shortcomings. The research was conducted in a small population and might not represent the majority of the population.

## 5. Conclusions

This research elucidates the complex dynamics of the digital divide as experienced by both healthcare providers and users throughout the COVID-19 pandemic, shedding valuable light on the implications for the future trajectory of digital health. By juxtaposing the perspectives of these two stakeholder groups, the study demonstrates that while digital health technologies hold transformative potential—particularly during crises—they also pose risks of deepening existing disparities if their implementation and integration are inadequately planned and supported.

In conclusion, the pandemic has exposed profound inequities within the realm of digital health access that must not be overlooked as healthcare systems continue to evolve. The findings from this study highlight an urgent need for inclusive strategies that balance technological innovation with accessibility. By addressing the barriers identified herein, stakeholders can fully harness the potential of digital health technologies to improve outcomes across diverse patient populations—especially during periods when accessible care is most critically needed.

## Figures and Tables

**Table 1:** Demographic data of healthcare employees (HEs) and consumers (Cs).

N	HEs N = 290	Cs N = 263
Gender		χ^2^ = 6.95, *p* = 0.138
Male	136	138
Female	154	125
Age		χ^2^ = 1.71, *p* = 0.202
20–30	17	21
31–40	27	34
41–50	78	66
51–60	99	99
61–70	69	43
Educational Level		χ^2^ = 51.75, *p* < 0.001
Higher Education	11	13
Nurse Assistant	34	59
Registered Nurse	88	106
Master of Science	139	75
Doctor of Philosophy	18	10
Foreign Language		χ^2^ = 5.04, *p* = 0.033
Yes	248	241
No	42	22

**Table 2:** Employment relationship of healthcare employees (HEs).

N	HEs N = 290
Type of employment relationship	
Full time	221
Part time	66
Practical training	3
Years in healthcare system	
0–9	84
10–19	54
20–29	93
>29	59
Workplace	
Hospitals	233
Primary healthcare	26
Other	31

## Data Availability

The original contributions presented in this study are included in the article. Further inquiries can be directed to the corresponding author(s).
